# Elucidation of the genetic architecture of water absorption capacity in hard winter wheat through genome wide association study

**DOI:** 10.1002/tpg2.20500

**Published:** 2024-08-27

**Authors:** Meseret A. Wondifraw, Zachary J. Winn, Scott D. Haley, John A. Stromberger, Emily E. Hudson‐Arns, R. Esten Mason

**Affiliations:** ^1^ Department of Soil and Crop Sciences Colorado State University Fort Collins Colorado USA; ^2^ USDA‐ARS Raleigh North Carolina USA

## Abstract

Water absorption capacity (WAC) influences various aspects of bread making, such as loaf volume, bread yield, and shelf life. Despite its importance in the baking process and end‐product quality, its genetic determinants are less explored. To address this limitation, a genome‐wide association study was conducted on 337 hard wheat (*Triticum aestivum* L.) genotypes evaluated over 5 years in multi‐environmental trials. Phenotyping was done using the solvent retention capacity (SRC) test with water (SRC‐water), sucrose (SRC‐sucrose), lactic acid (SRC‐lactic acid), and sodium carbonate (SRC‐carbonate) as solvents. Individuals were genotyped using genotyping‐by‐sequencing to detect single nucleotide polymorphisms across the wheat genome. To detect the genomic regions that underline the SRCs and gluten performance index (GPI), a genome‐wide association study was performed using six multi‐locus models using the *mrMLM* package in R. Adjusted means for SRC‐water ranged from 54.1% to 66.5%, while SRC‐carbonate exhibited a narrow range from 84.9% to 93.9%. Moderate to high genomic heritability values were observed for SRCs and GPI, ranging from *h* ^2 ^= 0.61 to 0.88. The genome‐wide association study identified a total of 42 quantitative trait nucleotides (QTNs), of which five explained over 10% of the phenotypic variation (*R* ^2^ ≥ 10%). Most of the QTNs were detected on chromosomes 1A, 1B, 3B, and 5B. Few QTNs, such as S1A_5190318, S1B_3282665, S4D_472908721, and S7A_37433960, were located near gliadin, glutenin starch synthesis, and galactosyltransferase genes. Overall, these results show WAC to be under polygenic genetic control, with genes involved in the synthesis of key flour components influencing overall water absorption.

## Core Ideas

- A total of 42 marker‐trait associations related to solvent retention capacity traits and gluten performance index were identified.
- Chromosomes 1A and 1B harbored the highest number of marker‐trait associations in this study.
- Most of marker‐trait associations identified in this study were frequently colocalized with gluten glycosyltransferase genes.

**Abbreviations**
BLUE: best Linear Unbiased estimate
FASTmrEMMA: fast multi‐locus random‐SNP‐effect efficient mixed‐model association
GPI: gluten performance index
GWAS: genome‐wide association study
LD: linkage disequilibrium
MAF: minor allele frequency
mrMLM: multi‐locus random‐SNP‐effect mixed linear model
NJ: neighbor‐joining
PC: principal component
PCA: principal component analysis
pKWmEB: integration of Kruskal–Wallis test with empirical Bayes under polygenic background control
QTL: quantitative trait locus
SNP: single nucleotide polymorphism
SRC: solvent retention capacity
WAC: water absorption capacity

## 1 INTRODUCTION

The bread‐baking quality of wheat (*Triticum aestivum* L.) is a primary determinant of the market price of the grain (Diriba et al., 2020). Water absorption capacity (WAC), which is a critical factor in the baking performance of wheat flour, refers to the amount of water a wheat flour can absorb to achieve optimum dough consistency for a desired baked product (Kweon et al., 2011). For hard winter wheat, a preferred wheat class to make bread and pizza dough, the WAC of flour influences bread production, both in terms of the bread's characteristics (Bushuk & Békés, 2002; Zghal et al., 2001) and the economy of its production (Bushuk & Békés, 2002; Pyler, 1979; Zghal et al., 2001). When flour has a high WAC, it can absorb more water, which provides two significant benefits for bakers. First, high WAC flour creates a larger volume of dough or bread per unit of flour, increasing bread yield. Bread yield can be maximized by increasing the amount of water in the formula without compromising bread quality and preventing water loss during baking (Puhr & D'Appolonia, 1992). Secondly, the use of high WAC flour may reduce the need for additional ingredients, which increases the costs of bread production.

The WAC of wheat flour is a complex trait that depends on various components present in the flour, including protein concentration, the degree of damaged starch, and pentosan/arabinoxylan concentration (Jelaca & Hlynka, 1971; Preston et al., 2001; Primo‐Martin et al., 2003; Rakszegi et al., 2014; Tipples et al., 1978). Other factors, such as kernel size (Morgan et al., 2000) and kernel hardness, can also affect WAC since they are related to the degree of damaged starch that occurs during the milling process (Pasha et al., 2010; Tipples et al., 1978). Harder grains are more resistant to milling, which results in more damaged starch. High levels of damaged starch and pentosan concentration lead to higher flour WAC (Kweon et al., 2011; Zghal et al., 2001).

The WAC of flour can be measured using methods such as the farinograph (AACC Approved Method 54‐21.02; AACC Intl., 2010) and mixograph (AACC Approved Method 54‐40.02; AACC Intl., 2010) tests. However, these procedures are time‐intensive and require substantial sample sizes. Furthermore, the Mixograph offers only a subjective measure of WAC (Ram et al., 2005). An efficient alternative is the solvent retention capacity (SRC) test. The SRC test uses four solvents, namely, water, lactic acid, sodium carbonate, and sucrose, to assess the functional contributions of flour components to overall product quality. The SRC test is faster and more efficient for determining a flour's WAC and understanding the roles of its components.

Genome‐wide association study (GWAS) is a genetic association mapping tool for identifying genomic regions related to complex traits in crops (Brachi et al., 2011; Zhao et al., 2011). Genome‐wide association studies identify quantitative trait nucleotides (QTNs), which can be used to elucidate the genetic architecture of a trait and to facilitate marker‐assisted selection (MAS). Previously, a few quantitative trait locus (QTL) mappings and genome‐wide association studies have reported the identification of genomic regions that control wheat dough rheological and baking traits, including WAC.

For instance, Ma et al. (2007) conducted a QTL mapping analysis on 92 doubled haploid populations of Australian wheat. The peak for WAC was located on chromosome 5A. Fox et al. (2013) conducted a QTL analysis for water absorption and flour yield using a doubled haploid population of 162 hard wheat individuals, and they reported QTLs on chromosome 4D. Similarly, Tsilo et al. (2013) performed composite interval mapping for farinograph water absorption and dough rheological properties using 139 hard red spring recombinant inbred lines (RILs). Six QTL on chromosomes 1A, 1B, 4B, 4D, and 5A were detected for farinograph water absorption. Campbell et al. (2001) reported several QTLs associated with damaged starch, alkaline water retention capacity (AWRC), and dough water absorption in a study using 78 RILs from a soft–hard wheat cross. For dough WAC, QTLs were identified on chromosomes 5A, 5B, and 5D for damaged starch and AWRC on chromosomes 4B, 4D, 5A, 5B, and 5D.

In addition, Xiao‐ling et al. (2023), using linkage mapping and association mapping in a RIL population consisting of 173 lines and an association panel of 205 common wheat varieties, identified 31 QTLs associated with SRC traits consisting of 57 QTNs, with two common chromosomal regions on chromosomes 1B and 4B found across both mapping populations. Similarly, Lou et al. (2021) conducted GWAS on 486 common wheat genotypes and reported 14 QTNs associated with WAC on chromosomes 2D, 3A, 3D, 5A, 5D, and 7A. Lastly, Navrotskyi et al. (2020) identified two single nucleotide polymorphisms (SNPs) on chromosome 4A associated with WAC in a study of 299 hard red winter wheat genotypes. Significant QTNs have been consistently reported on groups 1, 4, and 5 chromosomes across studies (Campbell et al., 2001; Lou et al., 2021; Navrotskyi et al., 2020), highlighting the crucial role of these chromosomes.

Despite WAC being a vital determinant of end use quality in hard wheat, most of the previous GWAS studies were on soft wheat classes, and studies are still limited to hard winter wheat. Therefore, this study was conducted to (1) elucidate the genetic architecture underlying WAC in hard winter wheat using multi‐locus GWAS models, which offer higher power to detect marker‐trait associations (MTAs) compared to previously reported single locus models; (2) identify candidate genes involved in WAC and related pathways, thereby providing valuable insights into the genetic mechanisms governing this trait in hard wheat; and (3) evaluate the impact of favorable allele combinations of significant MATs on each SRC trait which will help to determine the potential use in the breeding program.

**Core Ideas**

- A total of 42 marker‐trait associations related to solvent retention capacity traits and gluten performance index were identified.
- Chromosomes 1A and 1B harbored the highest number of marker‐trait associations in this study.
- Most of marker‐trait associations identified in this study were frequently colocalized with gluten glycosyltransferase genes.

## 2 MATERIALS AND METHODS

### 2.1 Plant materials and experimental designs

Note that 337 genotypes generated by the Colorado State University (CSU) wheat breeding program were used in this study. Hereafter, the term “genotype” will refer to both unreleased experimental lines and released cultivars. Genotypes were evaluated over a period of 5 years (2017–2021) in three independent breeding trials, namely, the CSU Elite Trial (ELITE) and the advanced yield nursery for conventionally derived genotypes (AYN) and doubled haploid‐derived genotypes (AYND). Trials were grown at CSU wheat breeding locations in the Great Plains wheat growing region of eastern Colorado. All trials were grown in farmer's fields, except for trials conducted at the Agriculture Research, Development, and Educational Center in Fort Collins, CO, and the United States Department of Agriculture Research Service Central Great Plains Center in Akron, CO. As such, the agronomic and crop management practices mirrored those used by the grower cooperators and varied according to the standard practices at each location.

Trials were arranged in resolvable, latinized row–column designs with partial replication following the methodology of John and Williams (1995) and Williams et al. (2011). In the CSU Elite trial, within a given location, half of the entries were replicated twice, and the other half were replicated once. A similar approach was used for the AYN and AYND, with approximately one‐seventh of the entries having a second replicate at any given location. Each plot was six rows, 1.5‐m wide and 3.7‐m long, seeded at approximately 1.73 million seeds ha^−1^. The grain was harvested from all six rows of each plot. A detailed description of the number of genotypes in each year–trial combination is given in Table 1.

**TABLE 1:** Number of observations and unique genotypes used by trial and year.

Year	Trial	Number of locations	Number of observations	Number of unique genotypes
2017	AYN	2	51	27
	AYND	3	91	70
	ELITE	2	70	35
2018	AYN	1	30	30
	AYND	1	18	18
	ELITE	4	155	50
2019	ELITE	6	785	100
2020	AYN	3	93	31
	AYND	3	116	53
	ELITE	4	600	100
2021	ELITE	4	590	100

Abbreviations: AYN, advanced yield nursery; AYND, advanced yield nursery of doubled haploid derived lines; ELITE, Colorado State University Elite Trial.

### 2.2 Phenotyping

To obtain white flour samples for SRC tests, 50 g grain samples from each genotype were processed by tempering to 14% moisture and milled using a Quadrumat Junior or modified Quadrumat Senior experimental mill from Brabender (South Hackensack). Flour moisture and protein concentration were determined using near‐infrared reflectance spectroscopy (NIR) (Foss DS2500 Feed and Forage Analyzer, Foss North America).

SRC tests were conducted on the milled flour samples. Two‐milliliter microcentrifuge tubes for each sample were labeled and pre‐weighed. To conduct the SRC tests, 200 ± 10 mg of flour from each sample was placed in each tube, and each of the four solvents (water, sucrose, lactic acid, and sodium carbonate) was used according to AACC International Approved Method 56‐11.02 (AACC Intl., 2010). A 50% weight/volume (w/v) sucrose solution was prepared by dissolving one part of sucrose crystals in one part of double‐distilled water, whereas the sodium carbonate and lactic acid solutions were prepared as 5% w/v. The flour and the solvent were vigorously mixed for 5 s to suspend the flour. The mixtures were then shaken for 20 min on a rotator, allowing the flour to mix well. Samples were then immediately centrifuged for 15 min at room temperature. After removal from the centrifuge, the supernatant was decanted, and the remaining pellet was left to dry for 10 min before weighing. To obtain the pellet weight, the initial tube weight was subtracted from the subsequent weight of the tube with the pellet. The SRC value (%) for each solvent was determined using the following formula:

$$\begin{array}{ccc} \mathrm{SRC}\;\left(\%\right) & = & \left[\frac{\left(\mathrm{Pellet}\;\mathrm{weight}(g\right)}{\left(\mathrm{Flour}\;\mathrm{weight}(g\right)}\;-\;1\right]\\ & & \;\times \left[\;\frac{\left(86\right)}{\left(100\;-\;\mathrm{Moisture}\;(\%\right)}\;\right]\times 100, \end{array}$$

where moisture (%) is the NIR‐determined moisture of the flour. The SRC test produced four SRC values for each sample, corresponding to the four solvents used. Gluten performance index (GPI), a more comprehensive measurement of the overall performance of flour glutenin in the context of other modulating networks of flour polymers (Kweon et al., 2011), was calculated according to Q. Zhang et al. (2007) using the following formula:

$$\begin{array}{ccc} \mathrm{GPI} & = & \left(\mathrm{SRC}-\mathrm{lactic}\;\mathrm{acid}\right)/\left(\mathrm{SRC}-\mathrm{sucrose}\right.\\ & & \left.+\;\mathrm{SRC}-\mathrm{carbonate}\right). \end{array}$$

### 2.3 Phenotypic data analysis

Across year–location–trial best linear unbiased estimates (BLUEs) were calculated in a one‐step analysis using *ASREML‐R* (Butler et al., 2009; R Core Team, 2013). The following mixed linear model was used to estimate BLUEs for each genotype:

$$y_{ijkl}=\mu +G_{i}+e_{j}+e_{j}:\;r_{k}+e_{j}:\;c_{l}+\varepsilon_{ijkl},$$

where $y_{ijkl}$ is the response variable for *i*th level of genotype, in the *k*th level of the row, and in the *l*th level of the column in the *j*th environment; μ is the overall mean; $G_{i\;}$ is the fixed effect of the *i*th genotype; $e_{j}$ the random effect of the *j*th environment; $e_{j}:r_{k}$ is the random interaction effect between the *j*th environment and the *k*th row; $e_{j}:c_{l}$ is the random interaction effect between the *j*th environment and the *l*th column; and $\varepsilon_{ijkl}$ is the residual error term $\varepsilon_{ijkl}∼N\left(0,\sigma_{\varepsilon }^{2}\right).$ The BLUEs calculated were then used as observed (phenotypic) values in the subsequent analysis.

Summary statistics (minimum, maximum, and average values) for SRCs and GPI were also calculated. Pearson correlation among pairs of BLUEs was done and visualized using the *psych* package in R (Revelle & Revelle, 2015).

To estimate variance components and calculate genomic heritability, genotypes were assigned as a random effect and analyzed with a mixed linear model using *ASREML‐R* (Butler et al., 2009). The following mixed linear model was used:

y=Xb+Zu+e,

where *X* and *Z* are known design matrices; ***b*** is a vector of fixed effects; ***u*** is a vector of random genetic effects with distribution $u∼N(0,\sigma_{g}^{2}K),$ where K is the kinship matrix and $\;\sigma_{g}^{2}$ is the genetic variance; ***e*** is a vector of random residuals with distribution $e∼N(0,\sigma_{e}^{2}I),$ where $\sigma_{e}^{2}$ is the residual variance and *I* is the identity matrix. Genomic heritability was then calculated using the following formula:

$$h^{2}=\frac{\sigma_{g}^{2}}{\sigma_{g}^{2}+\sigma_{e}^{2}}$$

where $\sigma_{g}^{2}$ is the genetic variance and $\sigma_{e}^{2}$ is the residual error associated with the trait.

### 2.4 Genotyping and quality control

Genomic DNA was extracted from 1‐week‐old leaves in a 96‐well format using the *MagMax14plant* DNA kit (Thermo Fisher Scientific), optimized for use with the KingFisher Flex magnetic particle processor equipped with a 96DW (96‐well‐deep well setting) particle head. DNA concentration was determined using PicoGreen (Thermo Fisher Scientific), which allowed normalizing DNA concentration to 20 ng µL^−1^ for library construction. Libraries were created using the *PstI‐MspI* restriction enzyme combination (Poland et al., 2012) and pooled together at a 384‐plex level.

Sequencing was performed on an Illumina HiSeq 2500 system at a core lab at the University of Illinois. SNP calling was performed using the *TASSEL GBSv2* pipeline (Glaubitz et al., 2014), with a 64‐base *k*‐mer length and a minimum *k*‐mer count of five. The Burrows–Wheeler aligner version 0.7.10 (H. Li & Durbin, 2009) was used to align reads to the International Wheat Genome Sequencing Consortium (IWGSC) RefSeq v1.0 Chinese Spring wheat reference genome (Appels et al., 2018).

The raw SNP data obtained from *TASSEL GBSv2* underwent a series of quality control steps. Initially, genotypes with over 50% missing calls and over 30% heterozygosity were filtered out. The filtration criteria were further tightened to retain only biallelic SNPs with a minor allelic frequency exceeding 5%, missing calls below 10%, and a maximum heterozygosity of 10%. Unaligned SNPs were discarded in the subsequent quality control phase. Imputation was then performed on the pruned dataset using the Beagle algorithm (Browning et al., 2018), resulting in a final set of 23,130 high‐quality SNPs for downstream analyses. The marker data used in this study represent a marker density of approximately 1.36 SNPs per 1 Mb. On average, the SNP distribution over 21 chromosomes was 1101 polymorphic SNPs per chromosome, but the distribution was nonuniform, with a minimum of 264 SNPs for chromosome 4D and a maximum of 1955 SNPs for chromosome 2B (Figure S1).

### 2.5 Population structure and linkage disequilibrium estimation

For purposes of GWAS analysis, both the Q‐matrix and kinship matrix were considered in the GWAS analysis to control the confounding effects of population stratification and individual relatedness on the associations between genetic markers and traits. To ascertain population structure, the optimal number of subpopulations (denoted as “*K*”) was determined using the entropy criterion. This method identifies the *K* value with the least cross‐validation error, as detailed by Alexander and Lange (2011) and Frichot et al. (2014). By testing a range of 2–20 potential populations and iterating each 10 times, the *sNMF* algorithm (implemented in the *LEA v 2.2.0* package in R) was employed. The outcome of this procedure was the ancestral coefficient (*Q*) for each genotype, based on the defined *K*. Additionally, the genetic relationship between individuals (kinship) was calculated using the *TASSEL* (Bradbury et al., 2007).

Additionally, as an alternative to the ancestral coefficient matrix (Q‐matrix), principal component analysis (PCA) was performed using the genotyping‐by‐sequencing marker data via the *prcomp* function in base R to analyze population structure (R Core Team, 2013). The optimal number of clusters (appropriate number of populations = *K*) was assigned based on the assumption of the majority rule from the *NbClust* package in R (Charrad et al., 2014). To support the population structure analyses, a neighbor‐joining (NJ) phylogenetic tree was constructed using *TASSEL 5.2* (Bradbury et al., 2007) and visualized using *interactive Tree of Life (iTOL) v3*, an online tool for display annotation and management of phylogenetic tree (Letunic & Bork, 2016).

To estimate linkage disequilibrium (LD) among the markers, which further helps to delineate the size of the region influenced by GWAS‐detected markers, pairwise LD values (*r* ^2^) were computed in *TASSEL* and plotted against physical distance (bp) in base R. The pattern of LD decay was determined as the distance where LD values are reduced to half of their maximum value (Remington et al., 2001).

### 2.6 Genome‐wide association study

To capture the genetic bases underlying WAC and contributing flour components, genome‐wide association analysis was performed for each of the four SRC traits and GPI using on six different multi‐locus GWAS models: Iterative modified‐sure independence screening EM‐Bayesian Lasso (*ISIS EM‐BLASSO*) (Tamba et al., 2017), multi‐locus random‐snp‐effect mixed linear model (*mrMLM*) (Wang et al., 2016), fast multi‐locus random‐snp‐effect efficient mixed‐model association (*FASTmrEMMA*) (Wen et al., 2018), integration of Kruskal–Wallis Test with empirical Bayes under polygenic background control (*pKWmEB*) (Ren et al., 2018), integration of least angle regression with empirical Bayes (J. Zhang et al., 2017), and fast multi‐locus random‐snp‐effect mixed linear model (*FASTmrMLM*) (Tamba & Zhang, 2018). All GWAS models were implemented using the *mrMLM* package in R (Wang et al., 2016).

Following the GWAS analysis, we applied the Bonferroni correction to adjust the significance thresholds for multiple testing. This method divides the standard alpha level (0.05) by the number of SNP tests performed (23,130), resulting in an adjusted significance threshold of 2.16 × 10^−6^, setting a stringent threshold for declaring statistical significance. The resulting *p*‐values were then transformed into −log_10_ values (5.7) to facilitate a more intuitive visualization and interpretation of the results. This adjustment ensures that our findings remain robust against the risk of type I errors, commonly heightened in studies involving a large number of comparisons.

Genotypes carrying one to six favorable alleles of significant MTAs were visualized using box plots to evaluate the effect of different combinations of favorable alleles. In addition, a stepwise linear regression analysis was conducted using the top six significant MTAs to understand the nature of their effects on the associated traits. Each individual QTN was modeled independently to assess its additive effect. Subsequently, interaction terms were introduced to the model to identify potential nonadditive effects, such as epistasis. The analysis utilized linear regression models with the significance of QTNs and their interactions determined based on *t*‐values and *p*‐values. If individual QTNs show a significant *p*‐value at a critical value of *α* < 0.05, then the effect is considered additive. If the interaction terms are significant, it suggests the presence of nonadditive effects.

### 2.7 Colocalization of QTNs and relevant genes for water absorption capacity

A colocalization analysis was done to reveal how closely significant QTNs were linked to genes previously reported to affect WAC either directly or indirectly through the synthesis of flour components such as gluten protein, starch, and arabinoxylans. The positions of the annotated genes involved in the synthesis of flour components and grain characteristics were retrieved from EnsemblPlants (https://plants.ensembl.org) with the IWGSC RefSeq v1.0 Chinese Spring (Appels et al., 2018). In addition, the KnetMiner (https://knetminer.com/cereals) wheat database was also used. Then, the positions of the genes were cross‐referenced with the positions of the significant MTAs identified in this study.

Using the calculated LD decay values of *r* ^2 ^= 0.3 as a threshold as a guideline (Figure S2), genes that are up to 4 Mb away from the significant QTNs were included as colocalized genes. To validate the associations between the identified genes and the QTNs within the specified search windows, an LD‐block analysis was performed using the *gpart* package in R (Kim et al., 2019). This analysis aimed to confirm the robustness of the genetic associations uncovered in the GWAS results.

## 3 RESULTS

### 3.1 Phenotypic evaluations and correlations

Summary statistics and heritability for SRCs and GPI are presented in Table 2. The SRC‐water ranged from 54.1% to 66.5%, with a mean of 59.0%. SRC‐sucrose ranged from 74.5% to 99.3%, with a mean of 87.6%. SRC‐lactic acid ranged from 93.9% to 123.2%, with a mean of 111.0%. GPI had a mean of 0.70 and ranged from 0.60 to 0.90. All SRCs and GPI showed moderate to high heritability (Table 2), with the lowest heritability (*h* ^2^ = 0.69 ± 0.04) recorded for SRC‐water and the highest heritability (*h* ^2^ = 0.88 ± 0.02) observed for SRC‐sucrose.

**TABLE 2:** Summary of phenotypic variation and heritability of solvent retention capacity traits and gluten performance index.

	Descriptive statistics			heritability	
Trait	Mean	Minimum	Maximum	*h* ^2^	SE
SRC‐water (%)	59.0	54.1	66.5	0.69	0.04
SRC‐sucrose (%)	87.6	74.5	99.3	0.88	0.02
SRC‐lactic acid (%)	111.0	93.9	123.2	0.82	0.05
SRC‐carbonate (%)	84.9	74.1	93.9	0.80	0.04
Gluten performance index	0.70	0.60	0.90	0.61	0.05

Abbreviations: *h* ^2^, broad sense heritability; SE, standard error; SRC, solvent retention capacity.

**FIGURE 1:**
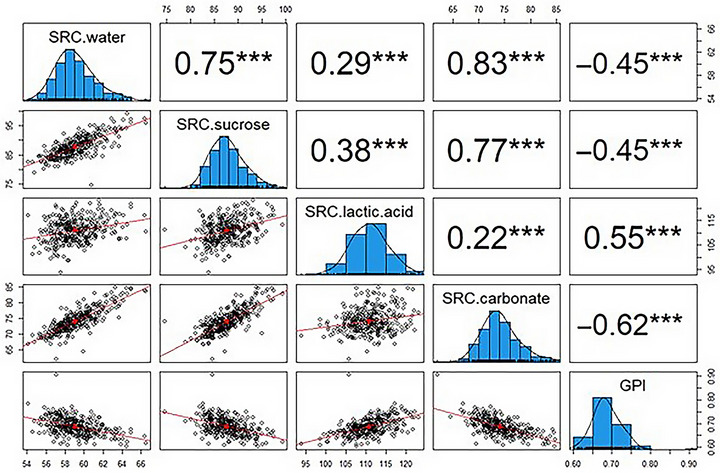
Pairwise correlation coefficients among traits studied in this study (solvent retention capacities [SRCs] and gluten performance index [GPI]) using best linear unbiased estimates (BLUEs) from across year–location analysis. The diagonal of the pair plot displays the frequency distribution for SRCs and GPI. The upper triangle shows Pearson's correlation coefficient (*R*) and the significance of the correlation for corresponding traits (***p ≤ 0.001). The lower triangle displays bivariate scatterplots with fitted lines.

Pearson's correlation coefficients ranged from *r* = −0.62 to *r* = 0.83; all identified correlations were highly significant (*p* < 0.001) (Figure 1). A strong positive correlation (*r* = 0.83; *p* < 0.001) was observed between SRC‐water, which is the overall WAC, and SRC‐carbonate, a proxy for the degree of damaged starch. Likewise, SRC‐lactic acid, which is a measure of flour gluten strength, had a positive correlation (*r* = 0.38; *p* < 0.001) with SRC‐sucrose, a measure of flour pentosan (arabinoxylan) concentration.

A negative correlation was observed between SRC‐water and GPI (*r* = −0.62, *p* < 0.001). Strong positive correlations were observed among SRC‐water, SRC‐sucrose, and SRC‐carbonate, with each correlation coefficient being above 0.70 (*p* < 0.001). Moderate correlations were observed between GPI and SRC‐water (*r* = −0.45; *p* < 0.001), SRC‐sucrose (*r* = 0.55; *p* < 0.001), and SRC‐lactic acid, as well as between SRC‐lactic acid and SRC‐water (*r* = 0.29; *p* < 0.001) and SRC‐sucrose (*r* = 0.38; *p* < 0.001). SRC‐lactic acid and SRC‐carbonate had a lower correlation (*r* = 0.22; *p* < 0.001).

### 3.2 Population structure

The population structure analysis revealed that the optimal number of subpopulations (*K*) was three, as determined using the entropy criterion to minimize cross‐validation error. This result was obtained using the *sNMF* algorithm implemented in the *LEA v 2.2.0* package in R. The ancestral coefficients (*Q*) for each genotype were calculated based on this *K* value. The resulting Q‐matrix, along with the kinship matrix from *TASSEL*, was included in the GWAS model to control confounding due to population stratification and ensure genuine association signals.

**FIGURE 2:**
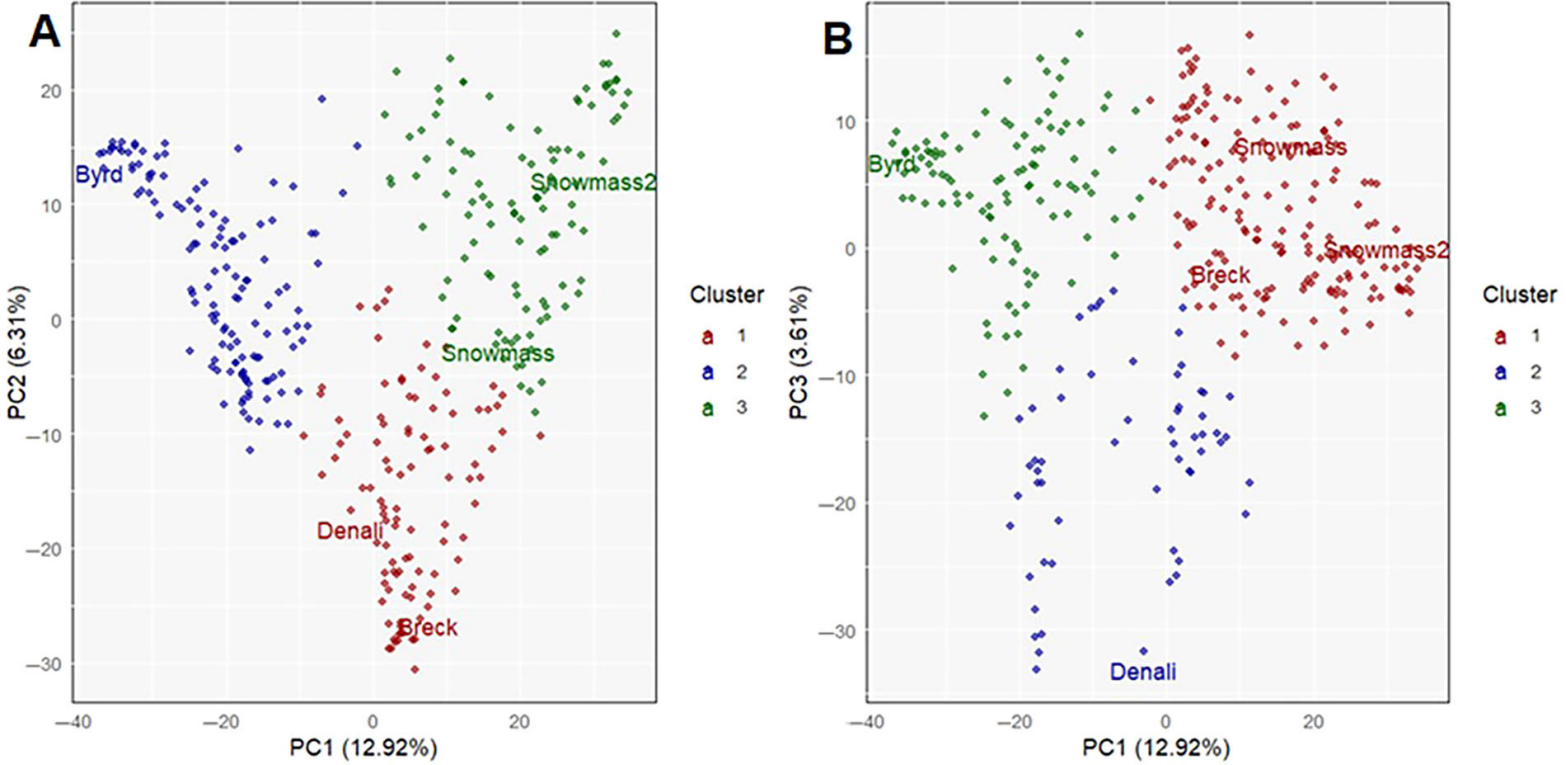
Scatter plot representing (A) the first and second (PC1 and PC2) and (B) the first and third (PC1 and PC3) principal components. The variance explained by each principal component is indicated in parentheses on each axis. Unique colors represent individual clusters.

In addition to Q‐matrix, PCA was conducted to discern and account for population stratification. The first three principal components (PCs) explained a combined 22.94% of the genetic variance (Figure 2A,B). In the PCA plot (A) PC1 versus PC2, three distinct clusters were evident. The clustering might be attributed to the genetic backgrounds of the genotypes from the three main parent varieties in the CSU wheat breeding program. Cluster one, mainly encompassing Byrd (PI 664257; Haley et al., 2012a) and its derivatives, was positioned toward the higher end of PC2 and across PC1. Cluster two, consisting largely of Denali (PI 664256; Haley et al., 2012b) and its derivatives, fell at lower values on both PC1 and PC2. Cluster three, predominantly including Snowmass (PI 658597; Haley et al., 2011) and Snowmass 2.0′ (PI 691605) and their derivative genotypes, occurred at the upper end of both PC1 and PC2. However, some genotypes did not align closely with any primary cluster, suggesting mixed genetic backgrounds. In PCA plot (B), PC1 versus PC3, genotypes from clusters one and three maintained positions similar to plot (A). However, cluster two genotypes displayed a more dispersed pattern, merging somewhat with the other clusters.

To further elucidate the population structure, an NJ phylogenetic tree was constructed. Similar groupings were revealed among genotypes, with three major clades exhibiting substantial overlap and shared ancestral backgrounds (Figure 2; Figure S3). The PCA and phylogenetic analyses provided clear visual representations of the genetic diversity and stratification within the studied genotypes. As such, a Q‐matrix and a kinship matrix were included in the GWAS models to minimize spurious genotype‐phenotype associations due to population substructure.

### 3.3 Genome‐wide association study

**FIGURE 3:**
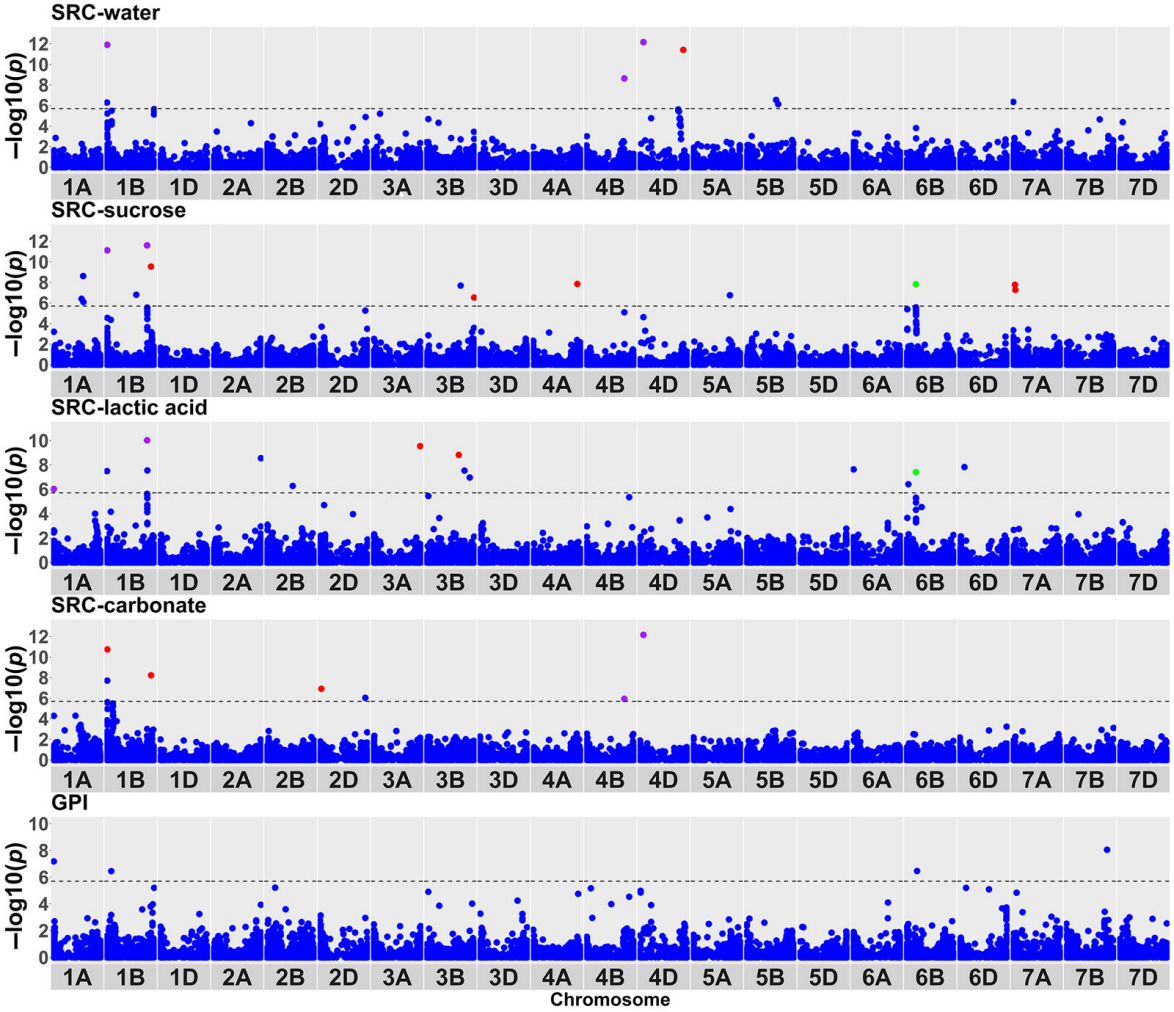
Manhattan plot illustrating −log_10_(*p*) values from a genome‐wide association study (GWAS) of solvent retention capacity (SRC) traits including SRC‐water, SRC‐sucrose, SRC‐lactic acid, SRC‐carbonate, and gluten performance index (GPI) displayed with its −log_10_(*p*) value on the *y*‐axis and its chromosomal position on the *x*‐axis. The dashed horizontal line marks the genome‐wide significance threshold of −log_10_(*p*) = 5.7. Points above this line highlight single nucleotide polymorphisms (SNPs) showing significant association with the SRC traits and GPI. The color coding of the points is detailed as follows: For marker‐trait associations (MTAs) identified by multiple models, only the highest −log_10_ *p*‐value will be displayed in the Manhattan plot and colored with red to indicate they were identified by more than one model. Purple dots represent MTAs identified by multiple models and associated with multiple traits; green dots signify MTAs identified by a single model but linked to multiple traits; and blue dots represent MTAs identified by one model and associated with one trait.

Considering each of the four SRC traits and the GPI and stringent Bonferroni‐adjusted significance threshold of 5.7 [−log_10_(*p*)], a total of 42 QTNs were identified across 17 chromosomes through six GWAS models. Some QTNs were identified by multiple models and associated with more than one trait, while others were specific to a single model and trait; for those identified by multiple models, only the highest −log_10_(*p*) is displayed in the Manhattan plot to avoid redundancy and confusion. Further details can be found in Table 3, Figure 3, and Table S1.

**TABLE 3:** Significant marker‐trait associations identified from genome wide association study for solvent retention capacity tests and gluten performance index using the best linear unbiased estimates.

QTN	Trait	Model	QTN effect	−log_10_(*p*)	*R* ^2^ (%)	MAF
S1A_353657879	SRC‐S	3	0.5985	6.04	2.75	0.42
S1A_373624388	SRC‐S	5	0.8315	8.61	7.46	0.25
S1A_377252109	SRC‐S	6	−0.6838	6.09	2.65	0.21
S1A_5190318	SRC‐L, GPI	5, 6	8.7003	6.02, 7.18	3.85, 6.41	0.21
S1B_3282665	SRC‐W	3, 4	0.5099	6.28, 6.36	1.58, 4.32	0.22
S1B_3767944	SRC‐L	3	1.6577	7.49	3.86	0.07
S1B_432872392	SRC‐S	4	0.9913	6.79	5.28	0.20
S1B_591353377	SRC‐S, SRC‐L	2, 4	−0.8406	10.02, 11.60	3.24, 5.32	0.41
S1B_595397803	SRC‐L	6	−0.9963	7.47	4.01	0.49
S1B_648546585	SRC‐S	5,6	0.8391	6.77, 9.53	5.34, 6.11	0.36
S1B_649243794	SRC‐C	2, 3, 4, 5	0.7959	6.65–8.23	3.17–5.25	0.49
S1B_66284969	GPI	2	0.2010	6.45	2.85	0.20
S1B_6922434	SRC‐W, SRC‐S	1, 2, 5	1.1445	6.93–11.90	4.97–11.18	0.22
S1B_7235112	SRC‐C	5	1.1559	7.71	9.51	0.22
S1B_8678756	SRC‐C	4, 5, 6	2.2190	6.25–10.74	6.18–13.15	0.14
S2A_787541163	SRC‐L	4	2.0009	7.54	2.79	0.06
S2B_444557348	SRC‐L	5	−0.9174	6.28	5.71	0.24
S2D_18933955	SRC‐C	3, 6	1.4964	5.85, 6.92	6.37, 8.37	0.10
S2D_624600035	SRC‐C	3	1.6264	6.04	6.26	0.07
S3A_741636006	SRC‐L	1, 2, 4, 6	1.2016	5.81–9.58	2.13–5.53	0.42
S3B_576577312	SRC‐L	2, 4	1.5428	6.56, 8.82	4.56, 6.08	0.15
S3B_609002613	SRC‐S	3	0.7828	7.69	3.59	0.22
S3B_677739256	SRC‐L	3, 5	1.2134	5.23, 7.53	5.62, 9.89	0.18
S3B_772483971	SRC‐L	6	0.7831	6.96	3.16	0.19
S3B_851549246	SRC‐S	4, 6	−1.2375	5.71, 6.53	2.62, 3.45	0.34
S4A_698874476	SRC‐S	2, 3, 4, 6	0.9649	5.93–7.85	3.89–5.64	0.23
S4B_535089330	SRC‐W, SRC‐C	2, 4, 6	1.4729	5.87–8.63	3.77–10.38	0.08
S4D_39790394	SRC‐W, SRC‐C	1, 2, 3, 4, 6	1.9685	8.36–12.16	6.56–11.73	0.10
S4D_472908721	SRC‐W	3, 5	1.9201	8.67, 11.41	6.53, 10.97	0.12
S5A_551174071	SRC‐S	6	−0.6417	6.75	2.37	0.29
S5B_444994793	SRC‐W	2	0.5057	7.36	5.63	0.38
S5B_444994802	SRC‐W	4	0.5707	6.54	7.19	0.38
S5B_477143477	SRC‐W	2	1.4020	6.13	3.77	0.49
S6A_6869286	SRC‐L	6	1.9555	7.63	3.5	0.35
S6B_147637875	SRC‐L, SRC‐S	6	0.9664	7.82	3.81	0.26
S6B_163949211	GPI	3	0.9390	6.46	3.32	0.13
S6B_30806573	SRC‐L	5	0.7901	6.41	3.4	0.44
S6D_49209864	SRC‐L	3	−0.7933	6.13	2.48	0.25
S7A_31213757	SRC‐S	3, 4	−0.7722	6.71, 7.77	2.70, 5.76	0.33
S7A_37433960	SRC‐S	5, 6	0.4199	7.14, 7.27	4.47, 7.41	0.34
S7A_3966595	SRC‐W	3	−0.3481	6.35	2.76	0.49
S7B_654488500	GPI	6	−0.8813	8.05	1.95	0.13

*Note*: Numbers before and after the underscore indicate the chromosome and position of the QTN on the chromosome, respectively. Model number: 1, *FASTmrEMMA*; 2, *FASTmrMLM*; 3, *ISISEM‐BLASSO*; 4, *mrMLM*; 5, *pKWmEB*; 6, *pLARmEB*.

Abbreviations: GPI, gluten performance index; −log10*p*, −log of *p*‐value; MAF, minor allele frequency; QTN, quantitative trait nucleotide; *R* ^2^, coefficient of determination indicating percent phenotypic variance explained; SRC, solvent retention capacity, SRC‐C using sodium carbonate as a solvent; SRC‐L using lactic acid as a solvent, SRC‐S using sucrose as a solvent, SRC‐W using water as a solvent.

Chromosome 1B had the highest number of QTNs (11), with associations for each of the four SRCs and GPI. Chromosome 3B had five QTNs associated with SRC‐sucrose and SRC‐lactic acid. Chromosome 1A had four QTNs, three associated with SRC‐sucrose and one associated with both SRC‐lactic acid and GPI. Chromosomes 5B, 6B, and 7A each had three QTNs associated with specific SRC phenotypes. Chromosomes 2D and 4D each had two QTNs associated with different SRCs, and the remaining chromosomes had a single QTN, while chromosomes 1D, 3D, 5D, and 7D had no significant associations. The highest number of QTNs were associated with SRC‐sucrose and SRC‐lactic acid (14 for each). Nine QTNs were associated with SRC‐water, seven were associated with SRC‐carbonate, and four were associated with GPI.

The most significant QTN was S4D_39790394, identified by five different models with −log_10_(*p*) values ranging from 8.36 to 12.16, which were associated with SRC‐water and SRC‐carbonate. Another significant QTN was S1B_6922434, with −log_10_(*p*) values ranging from 6.93 to 11.90, which was associated with both SRC‐water and SRC‐sucrose. A third significant QTN was S1B_591353377, which was associated with both SRC‐sucrose with −log_10_(*p*) of 10.02 and SRC‐lactic acid with −log_10_(*p*) value of 11.60 and minor allele frequency (MAF) of 0.41.

The proportion of phenotypic variance explained (*R* ^2^) by individual QTNs ranged from 1.58% to 13.15%, indicating small to moderate effects of individual QTNs (Table 3). The highest *R* ^2^ value was observed for QTN S1B_8678756, which was associated with SRC‐carbonate (explaining 13.15% phenotypic variance), while the minimum *R* ^2^ value was for QTN S1B_3282665 (1.58%), which was associated with SRC‐water. Five QTNs (S1B_8678756, S4B_535089330, S1B_6922434, S4D_39790394, and S4D_472908721) exhibited ≥10% *R* ^2^.

Several QTNs were associated with more than one SRC trait. Two QTNs, S1B_591353377 (*R* ^2^ = 3.24%–5.32%) and S6B_147637875 (*R* ^2^ = 3.13%–3.81%), were shared by SRC‐sucrose and SRC‐lactic acid. At the same time, S4B_535089330 (*R* ^2^ = 3.77%–10.38%) and S4D_39790394 (*R* ^2^ = 6.56%–7.73%) were shared by SRC‐water and SRC‐carbonate. Similarly, S1A_5190318 (*R* ^2^ = 3.85%–6.41%) was shared by SRC‐lactic acid and GPI, while S1B_6922434 (*R* ^2^ = 4.97%–11.18%) was shared by SRC‐water and SRC‐sucrose.

Some of the QTNs identified in this study, such as S2A_787541163 and S1B_3767944, both associated with SRC‐lactic acid, were rare in the population with MAFs of 0.06 and 0.07, respectively. Other QTNs, like S4B_535089330 with 0.08 MAF and S4D_39790394 with 0.20 MAF, both associated with SRC‐water and SRC‐carbonate, also had low frequencies. In contrast, QTNs such as S1B_595397803 (SRC‐lactic acid), S1B_649243794 (SRC‐carbonate), S5B_477143477, and S7A_3966595 (both SRC‐water) were found at much higher frequencies with MAFs of 0.49. The remaining significant QTNs were observed at moderate frequencies within the range between the highest and lowest MAFs observed.

In this study, several QTNs were captured by more than one model. Out of the 43 QTNs identified, 18 were captured by more than one model: 11 by two models, three by three models, three by four models, and one by five different models (Table 3; Table S2). The other 24 QTNs were identified by only one model (Table 3). For instance, QTN S1B_649243794 was identified by the *FASTmrEMMA*, *FASTmrMLM*, *ISISEM‐BLASSO*, *mrMLM*, and *pKWmEB* models, demonstrating associations with SRC‐water and SRC‐carbonate. Overall, both the *FASTmrMLM* and *ISISEM‐BLASSO* models detected QTNs associated with each trait, whereas the *pKWmEB* model did not detect any QTN for SRC‐water or GPI.

**FIGURE 4:**
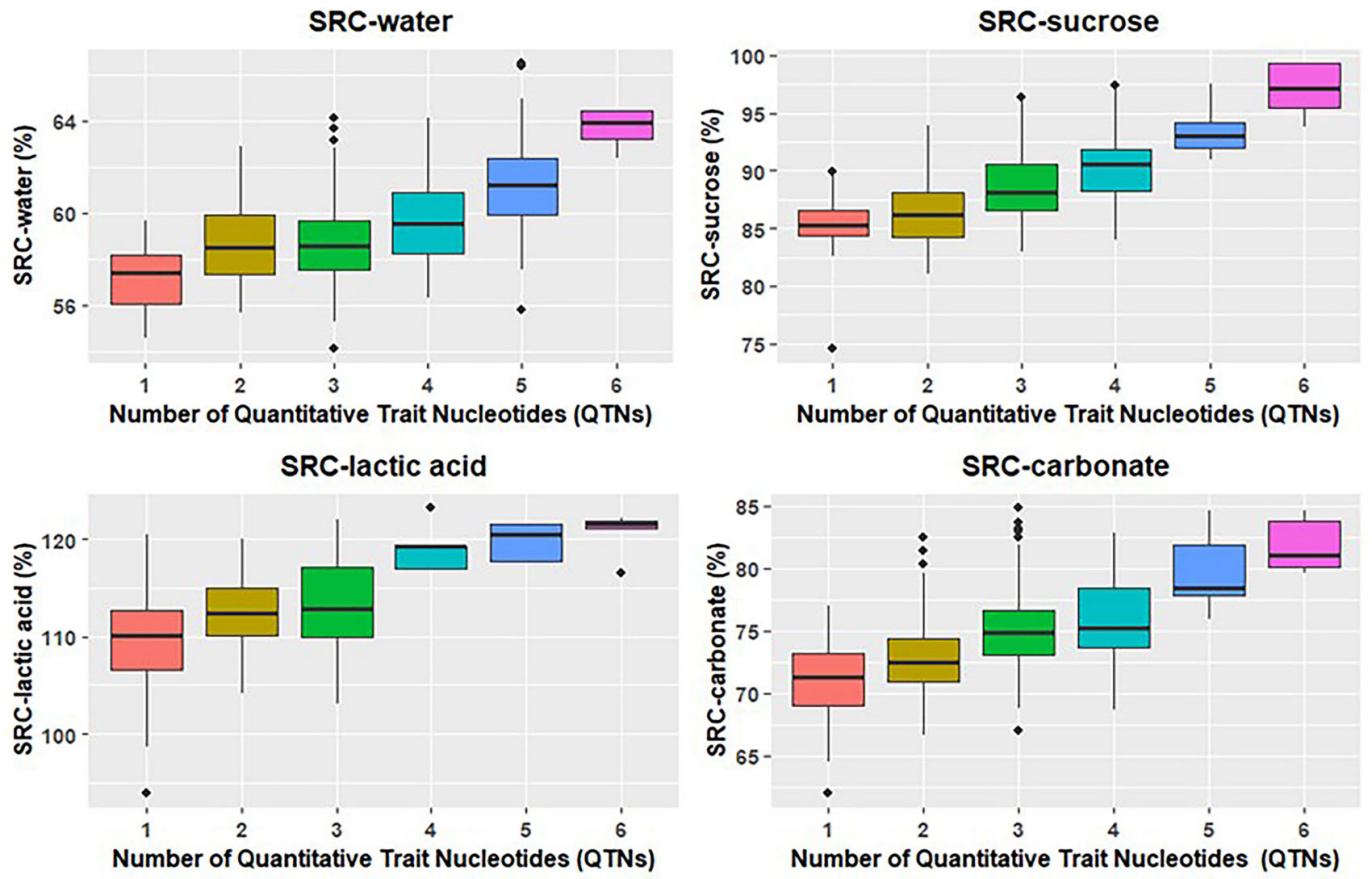
The cumulative effects of the top six significant markers associated with each solvent retention capacity (SRC) trait. Each box represents the interquartile range of the distribution across location best linear unbiased estimates, with the median indicated by a horizontal line within the box. Data points outside the box are considered outliers.

Genotypes were grouped based on the favorable alleles of the significant MTAs they carried to see the combined effects of the top six QTNs for each SRC trait. In general, with an increase in the number of favorable alleles present, the value of associated SRC traits also gradually increased (Figure 4). Genotypes carrying six favorable alleles for each SRC trait demonstrated a higher median value compared to genotypes carrying fewer than six QTNs. For all SRC traits, QTNs exhibited a more incremental additive effect with only small changes observed.

Stepwise linear regression analysis of all SRC traits showed that individual QTNs were significant (*α* < 0.05), confirming that traits were affected by the associated QTNs in an additive manner. Analysis revealed no epistatic interactions among the markers, as evidenced by nonsignificant interaction terms (Table S3).

### 3.4 Colocalization of QTNs and relevant genes for water absorption capacity

To validate the relevance of newly identified QTNs and to understand their relationship with established genetic markers, colocalization with known genes was investigated. Genes affecting WAC both directly and indirectly through flour components such as gluten, arabinoxylan, and damaged starch, as well as through grain hardness, were considered. Note that 43 annotated genes colocalized with 15 of the 42 significant QTNs identified in this study (Table 4), with key genes like low molecular weight‐glutenin subunit (*LMW‐GS*), gliadins, glycosyltransferase (*GT*), and *SS* located near the significant QTNs.

**TABLE 4:** Summary of colocalized quantitative trait nucleotides (QTNs) identified in this study and annotated genes.

Gene‐ID	Chrom	Significant QTNs	Traits	Distance (Mb)	Description
TraesCS1A02G007405	1A	S1A_5190318	SRC‐L, GPI	1.14	Gamma‐gliadin‐A3
TraesCS1A02G007400	1A	S1A_5190318	SRC‐L, GPI	1.15	Gamma‐gliadin‐A3
TraesCS1A02G007300	1A	S1A_5190318	SRC‐L, GPI	1.34	Gliadin
TraesCS1A02G007700	1A	S1A_5190318	SRC‐L, GPI	1.13	Gliadin
TraesCS1B02G010600	1B	S1B_3282665, S1B_3767944, S1B_6922434, S1B_7235112, S1B_8678756	All SRCs	1.72	Delta gliadin‐B1
TraesCS1B02G010500	1B	S1B_3282665, S1B_3767944, S1B_6922434, S1B_7235112, S1B_8678756	All SRCs	1.7	Delta gliadin‐B1
TraesCS1B02G010400	1B	S1B_3282665, S1B_3767944, S1B_6922434, S1B_7235112, S1B_8678756	All SRCs	1.68	Gamma‐gliadin‐3
TraesCS1B02G011000	1B	S1B_3282665, S1B_3767944, S1B_6922434, S1B_7235112, S1B_8678756	All SRCs	1.86	Gamma‐gliadin B
TraesCS1B02G011471	1B	S1B_3282665, S1B_3767944, S1B_6922434, S1B_7235112, S1B_8678756	All SRCs	2.09	Gliadin
TraesCS1B02G011300	1B	S1B_3282665, S1B_3767944 S1B_6922434, 1B_7235112, S1B_8678756	All SRCs	1.89	Gliadin
TraesCS1B02G009877	1B	S1B_3282665, S1B_3767944, S1B_6922434, S1B_7235112, S1B_8678756	All SRCs	1.56	Gliadin
TRITD1Av1G002790	1A	S1A_5190318	SRC‐L/GPI	0.96	LMW‐GS
TRITD1Av1G002360	1A	S1A_5190318	SRC‐L/GPI	0.14	LMW‐GS
TRITD1Av1G002310	1A	S1A_5190318	SRC‐L/GPI	0.24	LMW‐GS
TraesCS1A02G196100	1A	S1A_353657879	SRC‐S	0.64	Glycosyltransferase family 2
TraesCS1B02G023600	1B	S1B_7235112, 1B_8678756,	SRC‐C	3.73, 2.29	Glycosyltransferase
TraesCS1B02G023300		S1B_6922434, S1B7235112, S1B_8678756	SRC‐W, SRC‐S, SRC‐C	3.62,3.31,2.29	Glycosyltransferase
TraesCS1B02G083100	1B	S1B_66284969	GPI	0.73	Glycosyltransferase
TraesCS1B02G023700	1B	S1B_7235112, S1B_8678756	SRC‐C	3.89, 2.45	Glycosyltransferase
TraesCS1B02G080400	1B	S1B_66284969	GPI	3.56	Glycosyltransferase
TraesCS1B02G002100	1B	S1B_3282665	SRC‐W	1.59	Glycosyltransferase
TraesCS2B02G312100	2B	S2B_444557348	SRC‐L	2.81	Glycosyltransferase
TraesCS3B02G313900	3B	S3B_576577312	SRC‐L	2.23	Glycosyltransferase
TraesCS3B02G465600	3B	S3B_576577312	SRC‐L	3.46	Glycosyltransferase
TraesCS3B02G144500	3B	S3B_576577312	SRC‐L	2.73	Glycosyltransferase
TraesCS3B02G144900	3B	S3B_576577312	SRC‐L	2.71	Glycosyltransferase
TraesCS3B02G035000	3B	S3B_576577312	SRC‐L	1.89	Glycosyltransferase
TraesCS3B02G143100	3B	S3B_576577312	SRC‐L	2.05	Glycosyltransferase
TraesCS3B02G143300	3B	S3B_576577312	SRC‐L	2.19	Glycosyltransferase
TraesCS4D02G307700	4D	S4D_472908721	SRC‐W	2.96	Glycosyltransferase
TraesCS4D02G306800	4D	S4D_472908721	SRC‐W	2.01	Glycosyltransferase
TraesCS4D02G307000	4D	S4D_472908721	SRC‐W	2.04	Glycosyltransferase
TraesCS4D02G306900	4D	S4D_472908721	SRC‐W	2.03	Glycosyltransferase
TraesCS4D02G307300	4D	S4D_472908721	SRC‐W	2.21	Xyloglucan
TraesCS6A02G018200	6A	S6A_6869286	SRC‐L	1.99	Glycosyltransferase
TraesCS6A02G017900	6A	S6A_6869286	SRC‐L	1.88	Glycosyltransferase
TraesCS6A02G018000	6A	S6A_6869286	SRC‐L	1.92	Glycosyltransferase
TraesCS6B02G144400	6B	S6B_147637875	SRC‐L, SRC‐S	3.85	Glycosyltransferase
TraesCS7A02G057400	7A	S7A_31213757	SRC‐S	3.6	Glycosyltransferase
TraesCS7A02G002600	7A	S7A_3966595	SRC‐W	2.44	Glycosyltransferase
TraesCS7A02G015900	7A	S7A_3966595	SRC‐W	2.83	Glycosyltransferase
TraesCS1B02G368500	1B	S1B_595397803	GPI	3.48	Starch synthase, amyloplastic
TraesCS7A02G070100	7A	S7A_37433960	SRC‐S	1.66	Starch synthase, amyloplastic

Abbreviations: chrom, chromosome, GPI, gluten performance index; LMW‐GS, low molecular weight‐glutenin subunit; Mb, mega base; SRC, solvent retention capacity; SRC‐C, using sodium carbonate as a solvent; SRC‐L, using lactic acid as a solvent; SRC‐S, using sucrose as a solvent; SRC‐W, using water as a solvent.

Some QTNs, such as S1B_3282665, S1B_3767944, S1B_6922434, S1B_7235112, and S1B_8678756, were located 1.72 Mb away from *TraesCS1B02G010600*, which encodes the delta gliadin‐B1 (*Gli‐B1‐1*) gene, and 1.68 Mb away from *TraesCS1B02G010400*, which encodes the gamma‐gliadin‐3 (*Gli‐B1‐3*) gene. These genes and the QTN 1B_3767944 are in strong LD as evidenced in the Figure S4d. Similarly, S1A_5190318 (associated with SRC‐sucrose and SRC‐lactic acid) was located near *TraesCS1A02G007405* (1.14 Mb), *TraesCS1A02G007400* (1.15 Mb), *TraesCS1A02G007300* (1.34 Mb), and *TraesCS1A02G007700* (1.13 Mb), all encoding Gamma‐gliadin‐A3 (*Gli‐A1‐3*) genes. Additionally, S1A_5190318 is again situated near *TRITD1Av1G002790* (0.96 Mb), *TRITD1Av1G002360* (0.14 Mb), and *TRITD1Av1G002310* (0.24 Mb), known for encoding low molecular weight glutenin genes (*Glu‐B3*). LD block analysis in this region revealed that the QTN and the genes are in strong LD (∼*r* ^2^ = 0.9) (Figure S4a).

Additionally, on chromosome 1B, QTNs including S1B_3282665, S1B_3767944, S1B_6922434, S1B_7235112, and S1B_8678756, associated with various SRC traits, were found near *TraesCS1B02G010600*, *TraesCS1B02G010500*, *TraesCS1B02G010400*, and *TraesCS1B02G011000* (1.56–1.89 Mb) which also encode gliadins.

Other QTNs associated with all SRC traits and GPI, found on chromosomes 1B, 3B, 4D, 6A, 6B, and 7A, were located near genes encoding the *GT* gene family. For example, S1B_66284969 (associated with GPI) on chromosome 1B is situated 0.73 Mb away from *TraesCS1B02G083100*, which encodes *GT* genes. Similarly, S1B_3282665 (associated with SRC‐water) is 1.59 Mb from *TraesCS1B02G002100*, which encodes *GT* genes. Additionally, QTNs such as S3B_576577312 (associated with SRC‐lactic acid) are located near *TraesCS3B02G313900* (2.23 Mb), and S4D_472908721 (associated with SRC‐water) is near *TraesCS4D02G306800* (2.01 Mb), both encoding *GT* genes. S4D_472908721 is in strong LD with *TraesCS4D02G306800* (∼*r* ^2^ = 0.9) (Figure S4e). Likewise, S6A_6869286 (associated with SRC‐lactic acid) is near *TraesCS6A02G018000* (1.92 Mb), S6B_147637875 (associated with SRC‐sucrose and SRC‐lactic acid) is near *TraesCS6B02G144400* (3.85 Mb), and S7A_31213757 (associated with SRC‐sucrose) is near *TraesCS7A02G057400* (3.60 Mb), all encoding *GT* genes. The LD‐block analysis on chromosomes 1B and 7A showed that S1A_5190318 and S7A_31213757 are in strong LD (∼*r* ^2^ = 0.9) with the *GT* genes (Figure S4a,f), respectively). Moreover, two QTNs, S1B_595397803 (associated with GPI) located near *TraesCS1B02G368500* (3.48 Mb) and S7A_37433960 (associated with SRC‐sucrose) near *TraesCS7A02G070100* (1.66 Mb), encode for starch synthase (*SS*) and chloroplastic/amyloplastic genes.

## 4 DISCUSSION

Hard winter wheat is a preferred class for bread making due to its higher protein and gluten concentration, as well as its higher WAC (Mallory et al., 2012; Sapirstein et al., 2018). Genotypes included in this study exhibited lower average SRC‐water values (59%), which is low for bread‐making. SRC‐water measures the overall WAC of the flour and is influenced by various flour components, such as damaged starch, pentosans, and gluten proteins. Reduced levels of SRC‐water could be attributed to the absence of favorable alleles controlling these traits or, if present, to their low expression levels. Environmental stressors, which are common in the US Great Plains, including drought and extreme temperatures, can significantly impact the expression of genes associated with end use quality (Alsamman et al., 2021; Pandey et al., 2022). These stressors also affect the synthesis of glutenin proteins (Ronga et al., 2020; Wan et al., 2022) and arabinoxylans (Henry, 1986; Tremmel‐Bede et al., 2020).

Each of the evaluated SRC traits and GPI showed moderate to high heritability, in agreement with previous reports (Cabrera et al., 2015; Smith et al., 2011). High heritability suggests that the greater portion of the variation in these traits is due to genetic factors, and selection can result in genetic improvements (Bernardo, 2014). The strong positive correlation observed between SRC‐sucrose and SRC‐carbonate and the weak correlation observed between SRC‐carbonate and SRC‐lactic acid is consistent with other studies (Guttieri et al., 2001; Guzman et al., 2015). Furthermore, the strong positive correlation observed between SRC‐water and both SRC‐sucrose and SRC‐carbonate indicates that the higher the pentosans and damaged starch in the flour, the higher the overall WAC, consistent with previous reports (Colombo et al., 2008; Duyvejonck et al., 2011; Gaines, 2000; Guttieri et al., 2001; Guzman et al., 2015).

In this study, the observed sharing of QTNs among SRC traits and GPI, particularly between SRC‐lactic acid and SRC‐sucrose, SRC‐lactic acid and GPI, and SRC‐water and SRC‐carbonate, matches the observed phenotypic correlations between these traits. This alignment supports the previous findings of Guzman et al. (2015) and Jiang et al. (2017), reinforcing the genetic interconnectedness of these traits. Additionally, it suggests that the genes responsible for these traits could reside in the same region, or the same genes might affect both traits in a pleiotropic manner.

The genotypes, grouped based on the favorable alleles from the top six QTNs, showed an increase in the associated trait value as the number of alleles increased. In the stepwise regression analysis, the individual QTNs were significant, while the interaction terms were nonsignificant, suggesting that the marker effects predominantly reflect additive genetic influences and the absence of nonadditive effects. This additive effect indicates that each favorable allele contributes directly and independently to the phenotypic expression of SRC traits. This finding suggests that selecting these specific alleles in breeding programs would lead to improvements in SRC traits.

Significant QTNs identified in this study were distributed across 17 chromosomes. Chromosomes 1A, 1B, and 3B are significant hubs harboring many QTNs (20 out of 42) that are associated with all SRCs and GPI, suggesting these chromosomes are the crucial regions for WAC and flour components associated with WAC. Previous studies reported that significant QTNs and QTLs on chromosomes 1A and 1B are associated with WAC, grain hardness, grain weight, and milling traits (Aoun et al., 2022; Gaire et al., 2019; Garcia‐Santamaria et al., 2018; Ibba et al., 2021). At the same time, other studies have reported important QTLs in these regions for protein concentration, gluten concentration, starch concentration, and 1000‐kernel weight in both hard and soft wheat (Goel et al., 2019; Kerfal et al., 2010; McCartney et al., 2006). Current and previous studies suggest that, apart from the well‐known gluten genes, there may be additional genes on chromosomes 1A and 1B that play a significant role in regulating the end use quality of wheat.

The proximity of some QTNs on chromosomes 1A and 1B to the glutenin (*Glu‐B3*) and gliadin genes (*Gli‐A1‐3*, *Gli‐B1‐1*, *Gli‐B1‐9*, and *Gli‐B1‐3*) suggests their influence on wheat's WAC. The high molecular weight glutenin genes are located on chromosomes 1A, 1B, and 1D at the *Glu‐A1*, *Glu‐B1*, and *Glu‐D1* loci, whereas LMW glutenin genes are located on the short arms of these chromosomes at the *Glu‐A3*, *Glu‐B3*, and *Glu‐D3* loci (Payne et al., 1980; Shewry et al., 2003). Gliadin genes are primarily located on the short arms of group 1 chromosomes (1A, 1B, and 1D) at the *Gli‐A1*, *Gli‐B1*, and *Gli‐D1* loci. These loci encode gamma, delta, and omega gliadins. Additionally, the *Gli‐A2*, *Gli‐B2*, *and Gli‐D2* loci on the short arms of chromosomes 6A, 6B, and 6D encode for alpha and beta gliadins (Branlard et al., 2001; Payne et al., 1984; Shewry & Halford, 2002).

The QTN S1A_5190318, which is associated with SRC‐sucrose and SRC‐lactic acid, was located near low molecular weight glutenin (*Glu‐B3*) and gliadin (*Gli‐A1‐3*) genes. These genes were in strong LD (approximately *r* ^2^ = 0.9) with S1A_5190318, suggesting the association between them is not random and they can be inherited together. SRC‐sucrose is the proxy for pentosan (arabinoxylan) concentration and gliadin characteristics, while SRC‐lactic acid is for glutenin characteristics, mainly gluten quality (Dubat et al., 2019; Labuschagne et al., 2021). The location of QTN S1A_5190318 near the glutenin and gliadin genes indicates its potential as a genetic marker. This marker could be instrumental in wheat breeding programs, especially for traits influenced by gluten proteins, such as WAC.

Similarly, QTNs such as S1B_3282665, S1B_3767944, S1B_6922434, S1B_7235112, and S1B_8678756 were 0.14 Mb to 2.09 Mb away from the gliadin genes. These genes and the QTNs are in a strong LD (approximately *r* ^2^ = 0.7) range, suggesting a nonrandom association between these QTNs and the gluten genes, indicating a strong likelihood of co‐inheritance, implying that selecting gluten genes could enhance WAC in wheat.

Arabinoxylans/pentosans are cell wall polysaccharides that make up 65%–70% of the polysaccharide content in wheat and affect the WAC of the flour (Izydorczyk, 2021; Kosik et al., 2017). Genes such as glycosyltransferase families, xyloglucan endotransglucosylase/hydrolase (*XTH*) genes, and xylanases genes are reportedly involved in the regulation of pentosan synthesis and concentration (J. Li et al., 2021; Lovegrove et al., 2013). Glycosyltransferases are known for their involvement in the regulation of starchy and non‐starch polysaccharides, including arabinoxylans (J. Li et al., 2021; Lovegrove et al., 2013). Some of the QTNs in this study, such as S1B_7235112 (SRC‐carbonate), S1B_66284969 (GPI), S3B_576577312 (SRC‐lactic acid), S4D_472908721 (SRC‐water), and S6B_147637875 (SRC‐sucrose and SRC‐lactic acid), are located near *GT* genes. These genes are known for their role in the synthesis of arabinoxylans, which are crucial for WAC of a wheat flour. This proximity suggests that *GT* genes may also influence WAC. Downregulating the expression of *GT* gene families can reduce cell wall arabinoxylans in the endosperm by 40%–50% (Izydorczyk, 2021; Kosik et al., 2017).

In addition, QTNs from the current study, including S1B_649243794 (SRC‐carbonate), S1B_648546585 (SRC‐sucrose), and S1B_6922434 (SRC‐water and SRC‐sucrose), were colocalized with QTNs from previous reports such as S1B_642650411, S1B_651557718, and S1B_14665450 that are associated with total and water extractable arabinoxylans (Ibba et al., 2021). Those QTNs from current and previous reports were located 2–3 Mb away from each other. Given that the calculated LD decay (*R* ^2^ = 0.3) is very long (3.7 Mb), the current result and previously reported QTNs are still in strong LD, and both likely carry the same underlying variant.

Few QTNs, S1B_595397803 (associated with GPI) located near TraesCS1B02G368500 (3.48 Mb) and S7A_37433960 (associated with SRC‐sucrose) near TraesCS7A02G070100 (1.66 Mb), encode for SS and chloroplastic/amyloplastic genes. Starch synthase enzymes (*SS*) are responsible for the short chains of glucose polymers between branched clusters. These enzymes are important for the organization of the higher structure of starch granules (James et al., 2003). However, native starch (undamaged) absorbs relatively less water than damaged starch. In general, the proximal location of QTNs from the current study with annotated genes and previously reported QTNs and QTLs could be the conformation of the reliability of the MTAs in this study.

## 5 CONCLUSIONS

The multiple loci identified for SRCs and GPI traits across 17 chromosomes contribute smaller effects to the phenotype, suggesting that WAC is a polygenic, quantitatively inherited trait. The significant QTNs identified in this study, which appear to exhibit only additive effects in the studied population, provide an opportunity for utilization in a combined haplotype approach for MAS. This strategy offers a more pronounced effect on the phenotype than using individual QTNs in wheat breeding programs. The notable concentration of significant QTNs on chromosomes 1A, 1B, 3B, and 5B near genes involved in gliadin, glutenin, and starch synthesis underscores the potential of these genomic regions in improving WAC. Low molecular weight glutenin (*Glu‐B3*), gamma gliadins (*Gli‐A1‐3*, *Gli‐B1‐*3), and delta gliadins (*Gli‐B1‐1*) have been located near the significant QTNs, as have starch synthase genes (*SS*) on chromosomes 1A and 6B. These genes are considered potential candidates for affecting WAC in wheat. Breeders could leverage these QTNs for MAS to improve multiple traits simultaneously. Fine mapping and functional validation will be needed to pinpoint the causal polymorphisms responsible for the observed phenotypes and enable more precise introgression of beneficial haplotypes at these loci. In summary, the proximity of detected QTNs to previous QTNs and known genes provides insights and reveals key genomic regions likely to harbor functionally relevant variation for WAC.

## AUTHOR CONTRIBUTIONS

**Meseret A. Wondifraw**: Conceptualization; data curation; formal analysis; methodology; writing—original draft. **Zachary J. Winn**: Data curation; formal analysis; writing—review and editing. **Scott D. Haley**: Conceptualization; data curation; methodology; resources; supervision; writing—review and editing. **John A. Stromberger**: Data curation; resources. **Emily E. Hudson‐Arns**: Data curation; resources. **R. Esten Mason**: Funding acquisition; project administration; resources; supervision; validation; writing—review and editing.

## CONFLICT OF INTEREST STATEMENT

The authors declare no conflicts of interest.

## Supporting information

Supplemental Table S1. Significant marker‐trait associations identified in each chromosome. Abbreviations: solvent retention capacity (SRC), SRC‐water (SRC‐W), SRC‐sucrose (SRC‐S), SRC‐lactic acid (SRC‐L), SRC‐sodium carbonate (SRC‐C), and gluten performance index (GPI).
Supplemental Table S2. The number of Significant marker‐trait associations captured by each model. Abbreviations: SRC, solvent retention capacity; SRC‐W, using water as a solvent; SRC‐S, using sucrose as a solvent; SRC‐L, using lactic acid as a solvent; SRC‐C, using sodium carbonate as a solvent; GPI, gluten performance index.
Supplemental Table S3. Summary results from stepwise linear regression of the top six quantitative trait nucleotides for each SRC traits Abbreviations: SRC solvent retention capacity, Std.Error, Standard error
Supplemental Figure S1. Distribution of Single Nucleotide Polymorphisms (SNPs) used in the current study across Chromosomes 1A to 7D.
Supplemental Figure S2. Scatterplot depicting Linkage Disequilibrium (LD) decay among 337 hard winter wheat genotypes. The plot shows the squared correlation coefficient (*r* ^2^) against the genetic distance (in base pairs). The red line represents the fitted curve showing the decline of LD with increasing genetic distance. The blue horizontal line represents a predefined LD threshold. The point where the red curve intersects this blue line, indicating where LD has reduced to 50% of its maximum value, is marked on the x‐axis with a brown marker.
Supplemental Figure S3. The Neighbor‐Joining phylogenetic tree visualized using the Interactive *Tree of Life (iTOL)* software. The tree was derived from the analysis of dataset X and shows the genetic relationships among the studied taxa. Each branch represents a taxon, and the length of the branches indicates the genetic distance between them. Branch points, or nodes, represent hypothetical common ancestors.
Supplemental Figure S4. The LD (linkage disequilibrium) heatmap visualizes the strength of association between alleles at different loci on different wheat chromosomes. The color gradient, from red to yellow, indicates high to moderate linkage disequilibrium.

## Data Availability

The R Code used in this study can be in the data repository of the following GitHub account (https://github.com/MeseretAW/R‐codes‐mrMLM‐package_GWAS/blob/main/GWAS‐mrMLM_script.Rmd). The data used in our analysis can be made available upon request, subject to approval from all co‐authors of this work.
