# Supplementary material for: Elucidation of the genetic architecture of water absorption capacity in hard winter wheat through genome wide association study
Source: Plant Genome. 2024 Aug 27;17(3):e20500. doi: 10.1002/tpg2.20500 (PMC12807083; doi:10.1002/tpg2.20500)
Supplement: Supplementary file 1 [file TPG2-17-e20500-s001.docx]

# **Genome-Wide Association Study of Water Absorption Capacity in Hard Winter Wheat**

**Meseret A. Wondifraw^1*^, Zachary J. Winn^2^, Scott D. Haley^1^,** [**John A. Stromberger**](https://acsess.onlinelibrary.wiley.com/authored-by/Stromberger/John+A.)**^1^,** [**Emily E. Hudson**](https://acsess.onlinelibrary.wiley.com/authored-by/Hudson/Emily+E.)**^1^,** **R. Esten Mason^1*^**

1. Department of Soil and Crop Sciences, Colorado State University, Fort Collins, CO, USA 80523
2. United States Department of Agriculture Agricultural Research Service, Raleigh, NC, USA 27607

*Corresponding Author – meseret.wondifraw@colostate.edu

*Corresponding Author – [esten.mason@colostate.edu](mailto:esten.mason@colostate.edu)

**SUPPLEMENTAL MATERIALS**

Supplemental Table S1. Significant marker-trait associations identified in each chromosome.

| Chromosome | QTN detected | Traits | Models |
| --- | --- | --- | --- |
| 1A | 4 | SRCS-S, SRC-L | 3,5,6 |
| 1B | 11 | SRC-W, SRC-S, SRC-L, SRC-C, GPI | 1,2,3,4,5,6 |
| 2A | 1 | SRC-L | 5 |
| 2B | 1 | SRC-L | 3,6 |
| 2D | 2 | SRC-C | 3 |
| 3A | 1 | SRC-C | 1,2,4,6 |
| 3B | 5 | SRC-S, SRC-L | 3,5,6 |
| 4A | 1 | SRC-S | 2,3,4,6 |
| 4B | 1 | SRC-C | 2,3 |
| 4D | 2 | SRC-W, SRC-C | 1,2,3,4,6 |
| 5A | 1 | SRC-S | 6 |
| 5B | 3 | SEC-W | 2,4 |
| 6A | 1 | SRC-L | 6 |
| 6B | 3 | SRC-L, SRC-S, GPI | 3,5,6 |
| 6D | 1 | SRC-L | 3 |
| 7A | 3 | SRC-W, SRC-S | 3,4,5,6 |
| 7B | 1 | GPI | 6 |

Abbreviations: solvent retention capacity (SRC), SRC-water (SRC-W), SRC-sucrose (SRC-S), SRC-lactic acid (SRC-L), SRC-sodium carbonate (SRC-C), and gluten performance index (GPI).

Supplemental Table S2. The number of Significant marker-trait associations captured by each model.

| Model | Number of QTNs detected | Associated trait |
| --- | --- | --- |
| FASTmrEMMA | 3 | SRC-W, SRC-S, SRC-L, SRC-C |
| FASTmrMLM | 9 | SRC-W, SRC-S, SRC-L, SRC-C, GPI |
| ISIS EM-BLASSO | 14 | SRC-W, SRC-S, SRC-L, SRC-C, GPI |
| mrMLM | 12 | SRC-W, SRC-S, SRC-L, SRC-C |
| pKWmEB | 11 | SRC-S, SRC-L, SRC-C, GPI |
| pLARmEB | 16 | SRC-W, SRC-S, SRC-L, SRC-C, GPI |
| Total QTNs | 65 |  |
| Unique QTNs | 42 |  |
| QTNs identified by different models | 22 |  |

Abbreviations: SRC, solvent retention capacity; SRC-W, using water as a solvent; SRC-S, using sucrose as a solvent; SRC-L, using lactic acid as a solvent; SRC-C, using sodium carbonate as a solvent; GPI, gluten performance index.

Supplemental Table S3. Summary results from stepwise linear regression of the top six quantitative trait nucleotides for each SRC traits

| Coefficients:(SRC-water) | Estimate | Std.Error | t value | Pr(>\|t\|) |
| --- | --- | --- | --- | --- |
| Intercept | 56.67 | 0.28 | 200.11 | 0.00E+00 |
| S1B_3282665 | 0.79 | 0.22 | 3.59 | 3.82E-04 |
| S4D_39790394 | 3.15 | 0.51 | 6.22 | 1.53E-09 |
| S5B_477143477 | 0.56 | 0.19 | 2.97 | 3.16E-03 |
| S7A_3966595 | 0.34 | 0.19 | 1.77 | 7.71E-02 |
| S1B_662659763 | 1.38 | 0.23 | 5.97 | 6.01E-09 |
| S4D_472908721 | -0.25 | 0.48 | -0.52 | 6.06E-01 |
| Interaction of all QTNs | 0.51 | 0.77 | 0.67 | 5.05E-01 |
| Coefficients: (SRC-sucrose) | Estimate | Std.Error | t-value | Pr(>\|t\|) |
| Intercept | 85.63 | 0.52 | 166.03 | 2.740681e-319 |
| S1A_373624388 | -0.97 | 0.37 | -2.67 | 8.04E-03 |
| S1B_6922434 | 0.74 | 0.84 | 0.87 | 3.83E-01 |
| S1B_432872392 | 1.76 | 0.88 | 2.00 | 4.59E-02 |
| S1B_648546585 | -0.47 | 0.35 | -1.37 | 1.71E-01 |
| S3B_609002613 | 2.40 | 0.43 | 5.52 | 6.91E-08 |
| S4A_698874476 | 2.31 | 0.44 | 5.26 | 2.55E-07 |
| Interaction of all QTNs | 2.76 | 1.03 | 2.67 | 8.03E-03 |
| Coefficients:(SRC-lactic acid) | Estimate | Std.Error | t-value | Pr(>\|t\|) |
| Intercept | 108.76 | 0.42 | 257.39 | 0.00E+00 |
| S1B_3767944 | 3.84 | 0.95 | 4.04 | 6.56E-05 |
| S2A_787541163 | 3.18 | 1.05 | 3.02 | 2.73E-03 |
| S3A_741636006 | 1.84 | 0.47 | 3.88 | 1.25E-04 |
| S3B_576577312 | 0.61 | 0.69 | 0.89 | 3.76E-01 |
| S3B_677739256 | 3.75 | 0.61 | 6.09 | 3.10E-09 |
| S6A_6869286 | 0.37 | 0.47 | 0.79 | 4.31E-01 |
| Interaction of all QTNs | -0.04 | 2.53 | -0.02 | 9.88E-01 |
| Coefficients:(SRC-carbonate) | Estimate | Std.Error | t-value | Pr(>\|t\|) |
| Intercept | 68.68 | 0.69 | 99.447 | 2e-16 |
| S1B_7235112 | 1.63 | 0.38 | 4.31 | 2.13E-05 |
| S1B_649243794 | 1.70 | 0.36 | 4.71 | 3.71E-06 |
| S1B_654538334 | 0.23 | 0.41 | 0.56 | 0.017921 |
| S2D_18933955 | 1.89 | 0.45 | 4.16 | 4.15E-05 |
| S2D_624600035 | 1.47 | 0.49 | 2.98 | 0.00314 |
| S4D_39790394 | 4.96 | 0.61 | 8.10 | 1.09E-14 |
| Interaction of all QTNs | 2.4108 | 1.6127 | 1.495 | 0.13589 |

Abbreviations: SRC solvent retention capacity, Std.Error, Standard error

Supplemental Figure S1. Distribution of Single Nucleotide Polymorphisms (SNPs) used in the current study across chromosomes 1A to 7D.

Supplemental Figure S2. Scatterplot depicting Linkage Disequilibrium (LD) decay among 337 hard winter wheat genotypes. The plot shows the squared correlation coefficient (r^2^) against the genetic distance (in base pairs). The green line marks the threshold where LD drops to 50% of its maximum value. The LD decay value at this cutoff point is highlighted on the x-axis in green. This plot provides insights into the rate of LD decay, a crucial factor for genome-wide association studies in this population.

Supplemental Figure S3. The Neighbor-Joining phylogenetic tree visualized using the Interactive *Tree of Life (iTOL)* software. The tree was derived from the analysis of dataset X and shows the genetic relationships among the studied taxa. Each branch represents a taxon, and the length of the branches indicates the genetic distance between them. Branch points, or nodes, represent hypothetical common ancestors.

Supplemental Figure S4. The LD (linkage disequilibrium) heatmap visualizes the strength of association between alleles at different loci on different wheat chromosomes. The color gradient, from red to yellow, indicates high to moderate linkage disequilibrium.
